# Predictive Factors for Delayed Gastric Emptying After Pancreatoduodenectomy: A Swedish National Registry-Based Study

**DOI:** 10.1007/s00268-023-07175-2

**Published:** 2023-09-13

**Authors:** A. Hörberg Zdanowski, J. Wennerblom, J. Rystedt, B. Andersson, B. Tingstedt, Caroline Williamsson

**Affiliations:** 1 Department of Clinical Sciences Lund, Surgery, Lund University and Skåne University Hospital, Getingevägen 4, 221 85 Lund, Sweden; 2 Department of Surgery, Institute of Clinical Sciences Sahlgrenska Academy, Sahlgrenska University Hospital, University of Gothenburg, Gothenburg, Sweden

## Abstract

**Background:**

Delayed gastric emptying (DGE) is a common complication after pancreatoduodenectomy (PD). DGE causes prolonged hospital stay and a decrease in quality of life. This study analyzes predictive factors for development of DGE after PD, also in the absence of surgical complications.

**Method:**

Data from the Swedish National Pancreatic Cancer Registry for patients undergoing standard and pylorus preserving open PD from January 2010 until June 30, 2018, were collected. Data were analyzed in two groups, no DGE and DGE. A subgroup of patients with DGE but without surgical complications was compared to patients without DGE or any other surgical complication.

**Results:**

In total, 2503 patients were included, of which 470 (19%) had DGE. In the DGE group, 238 had other coexisting surgical complications and 232 had not. Postoperative pancreatic fistula (OR = 4.22, *p* < 0.001), surgical infection (OR = 1.44, *p* = 0.013), heart disease (OR = 1.32, *p* = 0.023) and medical complications (OR = 1.35, *p* = 0.025) increased the risk for DGE. A standard PD compared with pylorus preserving resection (OR = 1.69, *p* = 0.001) and a reconstruction with a pancreaticojejunostomy compared with a pancreaticogastrostomy (OR = 1.83, *p* < 0.001) increased the risk. For patients without surgical complications, a standard PD and reconstruction with pancreaticojejunostomy still increased the risk for DGE.

**Conclusion:**

DGE is more common after standard PD compared to pylorus preserving PD and after reconstruction with PJ compared to PG in this national cohort, both in the presence of other surgical complications as well as in the absence of other complications.

**Supplementary Information:**

The online version contains supplementary material available at 10.1007/s00268-023-07175-2.

## Introduction

The 5-year survival rate for pancreatic cancer is around 6% [1], but increases up to 28–38% if the tumor is radically resected and the patient completes adjuvant chemotherapy [2, 3]. The standard procedure for tumor resection for periampullary cancer is pancreatoduodenectomy (PD). Despite the evolution of surgical technique and standardization of perioperative care, major morbidity, such as postoperative pancreatic fistula (POPF) or intraabdominal abscesses, is still high and often reported to be 20–31% [4–7]. Mortality after PD in Sweden is 1.9% at 30-days [1]. About 30% never receive adjuvant chemotherapy due to postoperative complications [4].

One of the most common complications after PD is delayed gastric emptying (DGE), affecting 18–44% [8–11]. The International Study Group for Pancreatic Surgery (ISGPS) has established a globally acknowledged definition for DGE, which enables comparisons between hospitals and countries [12].

The causes for DGE are not entirely known, but are frequently associated with POPF or intraabdominal abscess [11, 13]. DGE is not lethal but results in a decreased quality of life, can prolong hospital stay or cause readmission, which in turn increases health care costs [12, 14].

The aim of this study is to analyze the incidence of and predictive factors for DGE after PD, based on data from the Swedish National Pancreatic and Periampullary Cancer Registry.

## Method

Data were retrieved from the Swedish National Pancreatic and Periampullary Cancer Registry, which was founded in 2009. It contains prospectively collected data on patients with tumors in the pancreatic or periampullary region, as well as all patients undergoing pancreatic surgery regardless of diagnosis. It is a national web-based secure registry, which was validated in 2016 and 2020. Until now it has enrolled over 16,000 patients [15]. The coverage rate on patients undergoing pancreatic surgery, resected or not, has been over 93% since 2010 [16].

Pancreatic surgery is centralized in Sweden, and in 2015, 93% of pancreatic resections were performed in six high-volume units [16]. The annual PDs are >150 at one unit, 75–150 at four sites and approximately 50 at one. Two of the mid-volume centers perform standard PD and pancreaticogastrostomy (PG) as reconstruction, two sites, mid- and lowest volume, perform pylorus preserving PD (PPPD) and pancreaticojejunostomy (PJ) and the final two sites perform standard PD with PJ. Most patients in Sweden undergo PD due to suspicion of malignancy in the periampullary region. In the postoperative histopathology report, the proportion of patients with chronic pancreatitis was 5% in 2019 [15].

In this study, patients 18 years or older who underwent PD or PPPD due to a periampullary tumor between January 1, 2010, and June 30, 2018, were included. During this time, only open approach for PD and PPPD was used in Sweden.

Demographic variables collected were sex, age, body mass index (BMI), smoking, weight loss (>10% of body weight) and comorbidities such as presence of diabetes or heart disease. BMI was calculated according to the definition of WHO [17]. Heart disease is defined as preoperative ongoing treatment with diuretics, antihypertensives, digoxin or anticoagulants. Furthermore, the American Society of Anesthesiologist score (ASA), presence of preoperative biliary drainage or neoadjuvant treatment were noted. Intraoperative factors included operation time, blood loss, transfusion, vascular resection and method of resection and reconstruction. Outcome and complications were registered and classified according to international standards: Clavien–Dindo classification [18], deep abscess, postoperative pancreatic fistula (POPF) [19], postpancreatectomy hemorrhage (PPH) [20], bile leakage, reoperation and 90-day mortality. The PPH of all grades were combined into one and recorded as present or not. From 2018, the registry changed to a compulsory grading of complications. For POPF, only clinically relevant POPF, grades B and C, were defined as fistulas [19]. Use of somatostatin analogues, length of stay and postoperative weight gain were noted. Clavien–Dindo ≥3a was considered as major complications. Postoperative weight gain was defined as ≥2 kg on postoperative day 1 since a 2 L intraoperative positive fluid balance may increase the risk for complications [21]. Medical complications were merged and included myocardial infarction, cardiac fibrillation, distal venous thrombosis, pulmonary embolism, pneumonia, urinary tract infection and kidney failure.

Delayed gastric emptying (DGE) was defined congruent with the definition set by the International Study Group of Pancreatic Surgery, as a prolonged need for gastric tube more than 4 days, reinsertion of a gastric tube after the third postoperative day or inability to tolerate solid food after one week [12]. The registry does not allow for a differentiation between DGE grades A, B and C before 2018, why this variable is categorized as “present” or “absent”.

The data were divided in two groups: patients with and without DGE, and these groups were compared. Furthermore, a subgroup analysis was performed on patients with isolated DGE, i.e. absent of other surgical complications such as POPF, PPH, bile leakage, surgical site infection, intraabdominal infection or any reoperation. This group was compared to the patients without any complications, DGE included.

Ethical approval for the study had been given by The Human Ethics Committee at Lund University, 2016/704, 24-09-2018.

## Statistical analysis

Descriptive statistics are presented as numbers and percentages, and median and interquartile range (IQR) as appropriate. Differences between groups were evaluated by the Chi-square analysis for categorical variables and Mann–Whitney *U* test for continuous variables. Multivariable analysis (MV) was performed using a stepwise backward logistic regression, with Hosmer–Lemeshow test for goodness of fit, of the variables with *p* < 0.25 in the univariate analysis for both all patients with DGE as well as for patients with DGE without any known surgical complication. The final model was tested and robust. Multiple imputation was therefore not used. Length of stay and reoperation were excluded from the MV analysis since they were considered to be secondary to DGE. Clavien–Dindo ≥3a was excluded from MV analysis since it was considered to reflect the severity of individual complications already in the analysis and severity of DGE in subgroup analysis. A *p *value of <0.05 was considered significant. Statistical analysis was conducted using SPSS^®^ version 27 (SPSS Inc^®^, Chicago, IL, USA).

## Results

### Patient and tumor features

A total of 2503 patients were included in the study, of which 470 (19%) developed DGE.

There were 1159 (46%) women, and the median age in total was 68 (IQR 62–74) years with no differences between the patients who developed DGE and not. Among the patients who developed DGE, BMI was significantly higher [25.3 (22.6–28.6) kg/m^2^ vs. 24.6 (22.3–27.5) kg/m^2^] and there was a higher proportion of heart disease (39 vs. 31%) in the DGE group, see Table 1.

**Table 1:** Demographics for patients with and without diagnosed DGE

	*n*	Total (*n* = 2503)	No DGE (*n* = 2033)	DGE (*n* = 470)	*p* value
Female sex	2503	1159	955 (47)	204 (43)	0.162
Age	2503	68 (62–74)	68 (62–73)	69 (62–74)	0.188
BMI	2405	24.7 (22.3–27.7)	24.6 (22.3–27.5)	25.3 (22.6–28.6)	**0.006**
Smoking	2399	423 (18)	355 (18)	68 (15)	0.170
Weight loss	2458	1279 (52)	1056 (53)	223 (48)	0.064
Diabetes	2481	484 (20)	402 (20)	82 (18)	0.238
Heart disease	2468	793 (32)	610 (31)	183 (39)	** <0.001**
Preop. biliary drainage	2479	1570 (63)	1283 (64)	287 (62)	0.386
Neoadjuvant treatment	2494	70 (3)	56 (3)	14 (3)	0.802
ASA ≥ 3	2478	610 (25)	485 (24)	125 (27)	0.256

Bold values indicate *p* < 0.05 and considered significant

Data presented as median (IQR) or numbers (%)

*BMI* body mass index, *ASA* physical status classification system by the American Society of Anesthesiologists

### Intraoperative data

Operation time was slightly, but significantly, shorter in the DGE group compared to no DGE, as shown in Table 2. There was a higher proportion of DGE among patients operated with standard PD compared to PPPD (20 vs. 15%, *p* = 0.025) and when reconstructed with PJ compared to PG (21 vs. 13%, *p* < 0.001).

**Table 2:** Intraoperative data for patients with and without DGE

	*n*	Total (*n* = 2503)	No DGE (*n* = 2033)	DGE (*n* = 470)	*p *value
Operation time (min)	2484	390 (329–451)	390 (330–452)	385 (309–451)	**0.043**
Intraoperative blood loss (ml)	2503	500 (300–900)	500 (300–900)	550 (300–985)	0.389
Vascular resection	2503	459 (18)	379 (19)	80 (17)	0.413
Intraoperative transfusion	2500	485 (19)	391 (19)	94 (20)	0.696
Type of operation	2503				**0.025**
PD		2011 (80)	1616 (79)	395 (84)	
PPPD		492 (20)	417 (21)	75 (16)	
Type of anastomosis	2490				** <0.001**
PJ		1767 (71)	1392 (69)	375 (80)	
PG		723 (29)	631 (31)	92 (20)	

Bold values indicate *p* < 0.05 and considered significant

Data presented as median (IQR) or numbers (%)

*PD* Pancreatoduodenectomy, *PPPD* pylorus preserving pancreatoduodenectomy, *PJ* Pancreaticojejunostomy, *PG* pancreaticogastrostomy

### Postoperative outcome

Postoperative course and outcome is demonstrated in Table 3. All complications were significantly more common in the DGE group, the intraabdominal drain was kept longer, and patients stayed longer in hospital. Somatostatin use was not associated with DGE. Surgical complications occurred in a total of 1172 (47%) patients. Of patients with DGE, 238 (10%) patients had other parallel surgical complications.

**Table 3:** Postoperative data for patients with and without DGE

	*n*	Total (*n* = 2503)	No DGE (*n* = 2033)	DGE (*n* = 470)	*p *value
Weight gain POD1 (≥ 2 kg)	1799	1121 (62)	855 (61)	266 (68)	** <0.001**
Drain (days)	2300	6 (4–9)	6 (4–8)	7 (5–10)	** <0.001**
Somatostatin^	2418	894 (36)	723 (37)	171 (37)	0.828
Clavien–Dindo ≥ 3a	2328	666 (27)	510 (25)	156 (33)	** <0.001**
Medical complication	2503	527 (21)	378 (19)	149 (32)	** <0.001**
Surgical complication	2325	1172 (47)			
POPF		245 (10)	123 (6)	122 (26)	** <0.001**
Surgical infection		424 (17)	289 (14)	135 (29)	** <0.001**
Biliary leakage		110 (4)	74 (4)	36 (8)	0.097
PPH		211 (8)	147 (7)	64 (14)	**0.001**
Length of stay (days)	2305	12 (8–17)	11 (8–15)	16 (11–23)	** <0.001**
90-day mortality	2305	85 (3)	65 (3)	20 (4)	0.254

Bold values indicate *p* < 0.05 and considered significant

Data presented as median (IQR) or numbers (%)

*POD 1* Postoperative day 1, *POPF* postoperative pancreatic fistula, *PPH* postpancreatectomy hemorrhage

^prophylactic somatostatin

Histopathology is demonstrated in Table 4. Benign and premalignant tumors, such as different cysts, were significantly more common among patients developing DGE.

**Table 4:** Histopathology report in the different DGE groups

	Total (*n* = 2503)	No DGE (*n* = 2033)	DGE (*n* = 470)	*p *value
Pancreas adenocarcinoma	1207 (48)	992 (49)	215 (46)	** <0.001**
Papillary cancer	295 (12)	242 (12)	53 (11)	0.466
Cholangiocarcinoma	218 (9)	178 (9)	40 (9)	0.624
Duodenal tumors	133 (5)	102 (5)	31 (7)	0.259
Benign tumors*	314 (13)	245 (12)	69 (15)	**0.001**
Chronic pancreatitis	92 (4)	64 (3)	28 (6)	** <0.001**
Other/not defined	75 (3)	55 (3)	20 (4)	** <0.001**

Bold values indicate *p* < 0.05 and considered significant

Data presented as actual numbers within each tumor histopathology entity and as proportion of each DGE group. 169 (7%) missing data in total cohort

*Benign tumors include premalignant tumors, such as serous and low-grade dysplasia mucinous cysts and IPMN

The logistic regression showed that POPF was the strongest independent factor for DGE. The only preoperative risk factor for DGE was having heart disease. The risk of DGE was higher following a standard PD resection as well as when reconstruction was made with PJ (Table 5).

**Table 5:** Univariable and multivariable analysis to identify predictive factors for DGE

	Event/total	Univariable analysis			Multivariable analysis		
		OR	95% CI	*p *value	OR	95% CI	*p *value
Female sex	1159/2503	1.16	0.94–1.41	0.160			
Age	2503/2503	1.01	1.00–1.02	0.276			
BMI	2405/2503	1.03	1.01–1.05	0.013			
Smoking	423/2399	0.82	0.62–1.09	0.170	0.71	0.52–0.97	0.032
Weight loss	1279/2458	0.83	0.68–1.01	0.065			
Diabetes	484/2481	0.85	0.66–1.11	0.238			
Heart disease	739/2468	1.47	1.19–1.81	<0.001	1.32	1.04–1.68	0.023
Operation time	2484/2503	1.00	1.00–1.00	0.005			
Type of operation PD	2011/2503	1.36	1.04–1.78	0.026	1.69	1.23–2.33	0.001
Intraoperative blood loss (ml)	2503/2503	1.00	1.00–1.00	0.117			
Type of anastomosis PJ	1767/2490	1.85	1.44–2.37	<0.001	1.83	1.37–2.45	<0.001
Weight gain POD1 (≥ 2 kg)	1121/1799	1.80	1.47–2.20	<0.001			
Drain (days)	2214/2300	1.03	1.02–1.04	<0.001			
Medical complication	527/2503	2.03	1.62–2.54	<0.001	1.35	1.04–1.74	0.025
POPF	245/2325	5.44	4.13–7.17	<0.001	4.22	3.06–5.82	<0.001
Surgical infection	424/2325	2.43	1.92–3.08	<0.001	1.44	1.08–1.92	0.013
Biliary leakage	110/2325	2.20	1.46–3.31	<0.001			
PPH	211/2325	2.02	1.48–2.76	<0.001			
Pancreas adenocarcinoma	1207/2334	0.80	0.65–0.98	0.030			
Benign tumors	314/2334	1.19	0.89–1.59	0.242			
Chronic pancreatitis	92/2334	1.85	1.18–2.93	0.008	1.82	1.07–3.09	0.028

Logistic regression by stepwise backward method, why only significant factors are shown in final step

*BMI* body mass index, *PD* pancreatoduodenectomy, *PG* pancreaticogastrostomy, *POD* postoperative day, *POPF* postoperative pancreatic fistula, *PPH* postpancreatectomy hemorrhage

### Patients with isolated DGE

After removing all patients with a surgical complication, except DGE, the subgroup contained 1570 patients. Of these, 232 [15% (or 9% totally)] had DGE. Demographics, perioperative and postoperative data were analyzed and showed that reconstruction with PJ still was a risk factor for DGE, see supplementary Tables S1–S3. Multivariable analysis, Table 6, revealed that heart disease, PD resection, PJ reconstruction, postoperative weight gain, and somatostatin independently increased the risk for DGE.

**Table 6:** Univariable and multivariable analysis to identify predictive factors for DGE when no surgical complications exist

	Event/total	Univariable analysis			Multivariable analysis		
		OR	95% CI	*p *value	OR	95% CI	*p *value
Age	1570/1570	1.01	0.99–1.03	0.237			
Female sex	751/1570	1.02	0.77–1.35	0.884			
BMI	1510/1510	1.02	0.98–1.05	0.363			
Weight loss	860/1546	0.90	0.68–1.12	0.438			
Smoking	274/1521	0.87	0.60–1.28	0.491			
Heart disease	504/1551	1.35	1.01–1.80	0.044	1.41	1.03–1.93	0.032
Operation time	1559/1559	1.00	1.00–1.00	0.095			
Vascular resection	303/1570	1.29	0.92–1.80	0.139			
Intraoperative blood loss (ml)	1570/1510	1.00	1.00–1.00	0.077			
Type of operation PD	1227/1570	1.31	0.92–1.88	0.136	1.60	1.59–2.42	0.026
Type of anastomosis PJ	1080/1560	1.48	1.07–2.05	0.017	1.56	1.06–2.31	0.025
Drain (days)	1555/1555	1.02	0.99–1.04	0.181			
Weight gain POD 1 (≥ 2 kg)	734/1570	1.55	1.17–2.05	0.002	1.45	1.06–1.98	0.020
Medical complication	296/1570	1.30	0.92–1.81	0.134			
Somatostatin	586/1562	1.25	0.94–1.66	0.129	1.73	1.23–2.42	0.001
Pancreas adenocarcinoma	865/1530	1.01	0.76–1.34	0.973			
Benign tumor	162/1530	0.85	0.52–1.37	0.493			s
Chronic pancreatitis	59/1530	2.44	1.37–4.37	0.003	2.54	1.33–4.88	0.005

Logistic regression by stepwise backward method, why only significant factors are shown in final step

*BMI* body mass index, *PD* pancreatoduodenectomy, *PG* pancreaticogastrostomy, *POD* postoperative day

## Discussion

Almost a fifth of all patients subject to PD experiences DGE postoperatively. DGE affects quality of life and leads to a prolonged hospital stay [11, 12, 14].

Many of the findings are not controversial. Obese people have a higher risk for complications after PD, and an association between high BMI and POPF has previously been shown [22, 23]. The median BMI was higher among the patients developing DGE, but it was not an independent risk factor. Robinson et al. [10] showed that a BMI over 35 was a risk factor for DGE, but it was correlated to the increased risk for POPF for obese patients.

Heart disease was more common in the DGE group. This factor has sparsely been studied. Robinson and colleagues [10] did not show any significant association between heart failure, coronary heart disease and DGE. In contrast, Welsch et al. [9] showed that heart failure in need of preoperative treatment was an independent risk factor for DGE. A large study including about 10,000 patients from Ellis et al. [24], on DGE without intraabdominal complications, showed that hypertension and chronic obstructive pulmonary disease increased the risk for DGE. Cardiac comorbidity might increase the risk for postoperative weight gain and cardiac or pulmonary comorbidity may result in later mobilization after surgery. The introduction of fast-track concepts in perioperative care with scheduled mobilization have shown less frequency of DGE [25]. Moreover, this study showed that smokers were less likely to experience DGE, as shown before [24].

Efforts have been made to find a solution for the problem with DGE in the surgical technique. Analysis of the Swedish data suggests that resection of the pylorus increased the risk for DGE. This is contradictory to several previous studies [26–28]. PPPD might suffer from a risk of postoperative pylorospasm secondary to denervation of the vagal nerve or ischemia [29]. Three randomized controlled trials (RCT) on pylorus resection or preservation and one meta-analysis did not find any differences in proportion of postoperative DGE though [30–33]. There is an ongoing RCT in Germany comparing standard PD and PPPD with DGE as the primary outcome that hopefully will enlighten this topic [34]. Until then, this present study aligns with others in giving contradicting results regarding the most beneficial resection technique considering DGE.

Several studies have analyzed different reconstruction techniques and DGE, also with conflicting results. In a RCT from 2005, Bassi et al. [35] showed that a PG results in less frequent DGE, similar to this study. However, others claim that PG has no effect on the risk of DGE at all [36–38] and some that DGE is more common in association with a PG [39]. A study from the Swedish registry found that PJ is significantly more associated with POPF than PG [6]. Similarly Bassi et al. [35] showed that postoperative collections were less common for PG, indicating that the rate of DGE is associated with POPF and intraabdominal infections. This, though, can only affect the 50% DGE patients, with concurrent complications and does not explain the similar results for the patients with isolated DGE.

A recent Cochrane update concluded that the various gastrojejunal reconstructions do not affect the proportion of DGE [40]. Still, some believe DGE is related to the duodenojejunostomy after PPPD, and a recently published study has shown decreased rates of DGE with a novel technique for duodenojejunostomy which allows for future widening of the anastomosis [41]. Data from minimally invasive PD show conflicting results regarding rate of DGE [42, 43], implying a need for further studies.

Operation time over five and a half hours has been suggested as a risk factor for DGE (21). This could not be supported by the result from this study. Vascular resection, perioperative transfusion or intraoperative bleeding were not associated with DGE, which is similar to previous reports [10, 13].

The group with DGE had more postoperative complications and prolonged hospital stay. Presence of a clinically relevant POPF gave more than four times increased risk for DGE, as shown previously [10, 11, 13]. The strong association between POPF and DGE also explains a higher BMI and longer requirement of drain in the DGE group [6, 44].

Medical complications independently increased the risk of DGE. Unfortunately, this study cannot distinguish between different types of medical complications. Pneumonia has been shown by others to result in an increased proportion of DGE [13]. On the other hand, another study showed that pneumonia did not significantly affect DGE, but respiratory failure and arrhythmia did [11].

The different histopathology outcomes did not affect DGE, except chronic pancreatitis, which was independently associated with increased numbers of DGE. This group composes a minority of all patients in this study, and previously no association has been found [13], why conclusions are difficult to make.

### DGE without surgical complications

The overall rate of isolated DGE was 9%, similar to a large American study [24]. A standard PD resection and a reconstruction with a PJ were both independent risk factors for DGE after adjusting for surgical complications. Further, a history of heart disease, postoperative weight gain and the use of somatostatin independently increased DGE. Prophylactic octreotide was demonstrated by Robinson et al. [10] to double the risk for DGE and an RCT on prophylactic somatostatin post-PD showed an increased number of DGE postoperatively [45]. In the study by Ellis et al. [24], age over 75 years, male sex, a PPPD and an operating time over seven hours increased the risk for isolated DGE, of which none were supported by this study.

The major strength of this study is the large sample size representing a national cohort of pancreatoduodenectomies in Sweden. The registry has been validated twice with high completeness of data and coverage over 90%.

This study has certain limitations inherent to the analysis of registry data. This study could only analyze variables in the registry, and several variables necessary for a reliable analysis of the surgical technique are not included in the registry. Pancreatic surgery in Sweden is centralized, with the aim to create high-volume centers with improved outcome. The annual report from the Swedish registry does not show any regional differences in outcome, but still type of resection and reconstruction varies among the centers as does individual technical skills and experience. This may have affected some results. However, the center and volume effects were not possible to analyze in the present dataset.

Another drawback is that the graded DGE variable, as described by the ISGPS, was introduced in the Swedish registry in 2018. Before 2018, DGE was denied or affirmed, making it impossible to distinguish between grades A, B and C.

## Conclusion

This study from the Swedish National Pancreatic and Periampullary Cancer Registry shows that heart disease, pancreatic fistula, medical complications, and a histopathology of chronic pancreatitis increase the risk of delayed gastric emptying after a pancreatoduodenectomy. A standard PD compared to PPPD, and reconstruction with pancreaticojejunostomy compared to pancreaticogastrostomy, increases the risk of delayed gastric emptying, both in the presence of other surgical complications as well as in the absence of other complications.

### Supplementary Information

Below is the link to the electronic supplementary material.

Supplementary file1 (DOCX 21 kb)

## References

[1] Committee S (2020) Swedish National Pancreatic and Periampullary Cancer Registry Annual Report 2020

[2] Strobel O, Hank T, Hinz U (2017). Pancreatic cancer surgery: the new R-status counts. Ann Surg.

[3] Maeda S, Moore AM, Yohanathan L (2020). Impact of resection margin status on survival in pancreatic cancer patients after neoadjuvant treatment and pancreatoduodenectomy. Surgery.

[4] Mackay TM, Smits FJ, Roos D (2020). The risk of not receiving adjuvant chemotherapy after resection of pancreatic ductal adenocarcinoma: a nationwide analysis. HPB (Oxford).

[5] McMillan MT, Christein JD, Callery MP (2016). Comparing the burden of pancreatic fistulas after pancreatoduodenectomy and distal pancreatectomy. Surgery.

[6] Williamsson C, Stenvall K, Wennerblom J (2020). Predictive factors for postoperative pancreatic fistula-a Swedish nationwide register-based study. World J Surg.

[7] Mackay TM, Gleeson EM, Wellner UF (2021). Transatlantic registries of pancreatic surgery in the United States of America, Germany, the Netherlands, and Sweden: comparing design, variables, patients, treatment strategies, and outcomes. Surgery.

[8] Atema JJ, Eshuis WJ, Busch OR (2013). Association of preoperative symptoms of gastric outlet obstruction with delayed gastric emptying after pancreatoduodenectomy. Surgery.

[9] Welsch T, Borm M, Degrate L (2010). Evaluation of the international study group of pancreatic surgery definition of delayed gastric emptying after pancreatoduodenectomy in a high-volume centre. Br J Surg.

[10] Robinson JR, Marincola P, Shelton J (2015). Peri-operative risk factors for delayed gastric emptying after a pancreaticoduodenectomy. HPB (Oxford).

[11] Mohammed S, Van Buren IG, McElhany A (2017). Delayed gastric emptying following pancreaticoduodenectomy: incidence, risk factors, and healthcare utilization. World J Gastrointest Surg.

[12] Wente MN, Bassi C, Dervenis C (2007). Delayed gastric emptying (DGE) after pancreatic surgery: a suggested definition by the international study group of pancreatic surgery (ISGPS). Surgery.

[13] Parmar AD, Sheffield KM, Vargas GM (2013). Factors associated with delayed gastric emptying after pancreaticoduodenectomy. HPB (Oxford).

[14] Ahmad SA, Edwards MJ, Sutton JM (2012). Factors influencing readmission after pancreaticoduodenectomy: a multi-institutional study of 1302 patients. Ann Surg.

[15] Steering-Committee (2019) Annual Report Swedish National Pancreatic and Periampullary Registry 2019

[16] Tingstedt B, Andersson B, Jonsson C (2019). First results from the Swedish national pancreatic and periampullary cancer registry. HPB (Oxford).

[17] World Health Organisation (1995) Physical status: the use and interpretation of anthropometry. Report of a WHO Expert Committee. World Health Organization Technical Report Series 845, pp 1–452 8594834

[18] Dindo D, Demartines N, Clavien PA (2004). Classification of surgical complications: a new proposal with evaluation in a cohort of 6336 patients and results of a survey. Ann Surg.

[19] Bassi C, Marchegiani G, Dervenis C (2017). The 2016 update of the international study group (ISGPS) definition and grading of postoperative pancreatic fistula: 11 years after. Surgery.

[20] Wente MN, Veit JA, Bassi C (2007). Postpancreatectomy hemorrhage (PPH): an international study group of pancreatic surgery (ISGPS) definition. Surgery.

[21] Fischer M, Matsuo K, Gonen M (2010). Relationship between intraoperative fluid administration and perioperative outcome after pancreaticoduodenectomy: results of a prospective randomized trial of acute normovolemic hemodilution compared with standard intraoperative management. Ann Surg.

[22] Ekstrom E, Ansari D, Williamsson C (2017). Impact of body constitution on complications following pancreaticoduodenectomy: a retrospective cohort study. Int J Surg.

[23] Akgul O, Merath K, Mehta R (2019). Postoperative pancreatic fistula following pancreaticoduodenectomy-stratification of patient risk. J Gastrointest Surg.

[24] Ellis RJ, Gupta AR, Hewitt DB (2019). Risk factors for post-pancreaticoduodenectomy delayed gastric emptying in the absence of pancreatic fistula or intra-abdominal infection. J Surg Oncol.

[25] Kuemmerli C, Tschuor C, Kasai M (2022). Impact of enhanced recovery protocols after pancreatoduodenectomy: meta-analysis. Br J Surg.

[26] Hackert T, Hinz U, Hartwig W (2013). Pylorus resection in partial pancreaticoduodenectomy: impact on delayed gastric emptying. Am J Surg.

[27] Zhou Y, Lin L, Wu L (2015). A case-matched comparison and meta-analysis comparing pylorus-resecting pancreaticoduodenectomy with pylorus-preserving pancreaticoduodenectomy for the incidence of postoperative delayed gastric emptying. HPB (Oxford).

[28] Jimenez RE, Fernandez-del Castillo C, Rattner DW (2000). Outcome of pancreaticoduodenectomy with pylorus preservation or with antrectomy in the treatment of chronic pancreatitis. Ann Surg.

[29] Kim DK, Hindenburg AA, Sharma SK (2005). Is pylorospasm a cause of delayed gastric emptying after pylorus-preserving pancreaticoduodenectomy?. Ann Surg Oncol.

[30] Hackert T, Probst P, Knebel P (2018). Pylorus resection does not reduce delayed gastric emptying after partial pancreatoduodenectomy: a blinded randomized controlled trial (PROPP Study, DRKS00004191). Ann Surg.

[31] Tran KT, Smeenk HG, van Eijck CH (2004). Pylorus preserving pancreaticoduodenectomy versus standard Whipple procedure: a prospective, randomized, multicenter analysis of 170 patients with pancreatic and periampullary tumors. Ann Surg.

[32] Klaiber U, Probst P, Strobel O (2018). Meta-analysis of delayed gastric emptying after pylorus-preserving versus pylorus-resecting pancreatoduodenectomy. Br J Surg.

[33] Busquets J, Martin S, Secanella L (2022). Delayed gastric emptying after classical Whipple or pylorus-preserving pancreatoduodenectomy: a randomized clinical trial (QUANUPAD). Langenbeck's Arch Surg.

[34] Renz BW, Adrion C, Klinger C (2021). Pylorus resection versus pylorus preservation in pancreatoduodenectomy (PyloResPres): study protocol and statistical analysis plan for a German multicentre, single-blind, surgical, registry-based randomised controlled trial. BMJ Open.

[35] Bassi C, Falconi M, Molinari E (2005). Reconstruction by pancreaticojejunostomy versus pancreaticogastrostomy following pancreatectomy: results of a comparative study. Ann Surg.

[36] Aroori S, Puneet P, Bramhall SR (2011). Outcomes comparing a pancreaticogastrostomy (PG) and a pancreaticojejunostomy (PJ) after a pancreaticoduodenectomy (PD). HPB (Oxford).

[37] Malleo G, Crippa S, Butturini G (2010). Delayed gastric emptying after pylorus-preserving pancreaticoduodenectomy: validation of International Study Group of Pancreatic Surgery classification and analysis of risk factors. HPB (Oxford).

[38] Eguchi H, Iwagami Y, Matsushita K (2020). Randomized clinical trial of pancreaticogastrostomy versus pancreaticojejunostomy regarding incidence of delayed gastric emptying after pancreaticoduodenectomy. Langenbeck's Arch Surg.

[39] Hayama S, Senmaru N, Hirano S (2020). Delayed gastric emptying after pancreatoduodenectomy: comparison between invaginated pancreatogastrostomy and pancreatojejunostomy. BMC Surg.

[40] Huttner FJ, Klotz R, Ulrich A (2022). Antecolic versus retrocolic reconstruction after partial pancreaticoduodenectomy. Cochrane Database Syst Rev.

[41] Machado MA, Mattos BV, Lobo Filho MM (2023). A new technique of duodenojejunostomy may reduce the rate of delayed gastric emptying after pylorus-preserving pancreatoduodenectomy: the growth factor technique (with video). Surg Oncol.

[42] Liu Q, Zhao Z, Zhang X (2023). Perioperative and oncological outcomes of robotic versus open pancreaticoduodenectomy in low-risk surgical candidates: a multicenter propensity score-matched study. Ann Surg.

[43] Mao SH, Shyr BS, Chen SC (2022). Risk factors for delayed gastric emptying in pancreaticoduodenectomy. Sci Rep.

[44] Ellis RJ, Brock Hewitt D, Liu JB (2019). Preoperative risk evaluation for pancreatic fistula after pancreaticoduodenectomy. J Surg Oncol.

[45] Cao Z, Qiu J, Guo J (2021). A randomised, multicentre trial of somatostatin to prevent clinically relevant postoperative pancreatic fistula in intermediate-risk patients after pancreaticoduodenectomy. J Gastroenterol.

