# Supplementary material for: Predictive Factors for Delayed Gastric Emptying After Pancreatoduodenectomy: A Swedish National Registry-Based Study
Source: World J Surg. 2023 Sep 13;47(12):3289–97. doi: 10.1007/s00268-023-07175-2 (PMC10694105; doi:10.1007/s00268-023-07175-2)
Supplement: Supplementary file 1 — Supplementary file1 (DOCX 21 kb) [file 268_2023_7175_MOESM1_ESM.docx]

**Supplementary tables, cohort without surgical complications.**

**Table S1. Demographics for subgroup-analysis of patients with and without DGE and no other surgical complications.**

|  | **n** | **Total**  **(n=1570)** | **No DGE**  **(n=1338)** | **DGE**  **(n=232)** | **p-value** |
| --- | --- | --- | --- | --- | --- |
| **Female sex** | 1570 | 751 (48) | 639 (48) | 112 (48) | 0.884 |
| **Age** | 1570 | 68 (62-74) | 68 (62-73) | 69 (62-75) | 0.153 |
| **BMI** | 1510 | 24.4 (22.1-27.2) | 24.4 (22.1-27.0) | 24.4 (21.9-28.0) | 0.328 |
| **Smoking** | 1521 | 274 (18) | 238 (18) | 36 (16) | 0.491 |
| **Weight loss** | 1546 | 860 (56) | 738 (56) | 122(53) | 0.438 |
| **Diabetes** | 1558 | 320 (21) | 276 (21) | 44 (19) | 0.567 |
| **Heart disease** | 1551 | 504 (33) | 416 (32) | 88 (39) | **0.043** |
| **Preop biliary drainage** | 1556 | 1036 (67) | 1883 (67) | 153 (67) | 0.936 |
| **Neoadjuvant treatment** | 1565 | 43 (3) | 35 (3) | 8 (3) | 0.479 |

Data presented as numbers (%) or median (IQR).
BMI – body mass index, ASA – American Society of Anesthesiologists classification system

**Table S2. Perioperative data for subgroup-analysis of patients with and without DGE and no other surgical complications.**

|  | **n** | **Total**  **(n=1570)** | **No DGE**  **(N=1338)** | **DGE** **(N=232)** | **p-value** |
| --- | --- | --- | --- | --- | --- |
| **Operation time (min)** | 1559 | 390 (330-450) | 390 (330-450) | 385 (318-450) | 0.358 |
| **Intraoperative blood loss (ml)** | 1570 | 500 (300-900) | 500 (300-900) | 500 (250-900) | 0.968 |
| **Vascular resection** | 1570 | 303 (19) | 250 (19) | 53 (23) | 0.138 |
| **Intraoperative transfusion** | 1569 | 287 (18) | 240 (18) | 47 (20) | 0.382 |
| **Type of operation** | 1570 |  |  |  | 0.135 |
| **PD** |  | 1227 (78) | 1037 (78) | 190 (82) |  |
| **PPPD** |  | 343 (22) | 301 (23) | 42 (18) |  |
| **Type of anastomosis** | 1560 |  |  |  | **0.017** |
| **PJ** |  | 1080 (69) | 906 (68) | 174 (76) |  |
| **PG** |  | 480 (31) | 425 (32) | 55(24) |  |

**Table S3. Postoperative data for subgroup-analysis of patients with and without DGE and no other surgical complications.**

|  | **n** | **Total**  **(n=1570)** | **No DGE**  **(N=1338)** | **DGE**  **(N =232)** | **p-value** |
| --- | --- | --- | --- | --- | --- |
| **Weight gain POD1 (≥2 kg)** | 1570 | 734 (47) | 604 (45) | 130 (56) | **0.002** |
| **Drain (days)** | 1555 | 5 (4-8) | 5 (4-7) | 6 (4-8) | **0.002** |
| **Somatostatin** | 1562 | 586 (38) | 489 (37) | 97 (42) | 0.128 |
| **Clavien Dindo ≥3a** | 1570 | 59 (4) | 39 (3) | 20 (9) | **<0.001** |
| **Medical complication** | 1570 | 296 (19) | 244 (18) | 52 (22) | 0.133 |
| **Length of stay (days)** | 1557 | 13 (8-14) | 10 (8-14) | 14 (9-18) | **<0.001** |
| **90-day mortality** | 1570 | 22 (1) | 20 (2) | 2 (1) | 0.449 |

Data presented as numbers (%) or median (IQR). POD 1 - Postoperative day
